# Altered connectivity of the dorsal and ventral visual regions in dyslexic children: a resting-state fMRI study

**DOI:** 10.3389/fnhum.2015.00495

**Published:** 2015-09-10

**Authors:** Wei Zhou, Zhichao Xia, Yanchao Bi, Hua Shu

**Affiliations:** ^1^State Key Laboratory of Cognitive Neuroscience and Learning and IDG/McGovern Institute for Brain Research, Beijing Normal UniversityBeijing, China; ^2^Center for Collaboration and Innovation in Brain and Learning Sciences, Beijing Normal UniversityBeijing, China; ^3^Beijing Key Lab of Learning and Cognition, Department of Psychology, Capital Normal UniversityBeijing, China

**Keywords:** dyslexia, visual attention deficit, dorsal visual region, resting-state

## Abstract

While there is emerging evidence from behavioral studies that visual attention skills are impaired in dyslexia, the corresponding neural mechanism (i.e., deficits in the dorsal visual region) needs further investigation. We used resting-state fMRI to explore the functional connectivity (FC) patterns of the left intraparietal sulcus (IPS) and the visual word form area (VWFA) in dyslexic children (*N* = 21, age *mean* = 12) and age-matched controls (*N* = 26, age *mean* = 12). The results showed that the left IPS and the VWFA were functionally connected to each other in both groups and that both were functionally connected to left middle frontal gyrus (MFG). Importantly, we observed significant group differences in FC between the left IPS and the left MFG and between the VWFA and the left MFG. In addition, the strengths of the identified FCs were significantly correlated with the score of fluent reading, which required obvious eye movement and visual attention processing, but not with the lexical decision score. We conclude that dyslexics have deficits in the network composed of the prefrontal, dorsal visual and ventral visual regions and may have a lack of modulation from the left MFG to the dorsal and ventral visual regions.

## Introduction

Learning to read fluently is one of the most important tasks for children. Fluent reading requires the precise integration of vision, attention, eye movements, and linguistic processes. While the auditory-phonological processing deficits have gradually become the predominant explanation for developmental dyslexia in the last few decades (Snowling, 2001; Goswami, 2003, 2011; Ramus, 2003; Ramus and Szenkovits, 2008; Gabrieli, 2009; Shamma and Micheyl, 2010; for reviews), the role of visual processing in dyslexia remains compelling. Recently, there is emerging evidence from behavioral studies that visual attention skills are impaired in dyslexia (Hari and Renvall, 2001; Facoetti, 2004, 2012; Valdois et al., 2004; Vidyasagar and Pammer, 2010; Gori and Facoetti, 2014; for reviews). According to previous functional magnetic resonance imaging (fMRI) studies, visual attention, and eye movement skills have been reported to be associated with the dorsal visual region in the brain (e.g., Corbetta, 1998; Corbetta et al., 1998). Until recently, however, only a small number of fMRI studies (e.g., Peyrin et al., 2011; Lobier et al., 2014) have examined the dysfunction of the dorsal visual region in dyslexia, whereas there have been considerable studies concentrating on deficits in the ventral visual region, such as the occipitotemporal cortex (OTC; McCandliss et al., 2003; Shaywitz and Shaywitz, 2005; Richlan et al., 2009; for reviews). The current study used resting-state fMRI to investigate whether functional connectivity (FC) with the seed in the ventral visual region or dorsal visual region is altered in children with dyslexia.

The visual attention deficit theory of dyslexia highlights the importance of visual attention factors in reading (Vidyasagar and Pammer, 2010). Typically in fluent reading, a given sentence consists of multiple words and each word consists of multiple letters in a spatial sequence. Due to the constraint of humans' attention resources and fovea fields, readers need efficient attention shifting/allocating, parafoveal processing, and eye movement mechanisms to be engaged in relevant targets (e.g., to serially select the positions of letters or words; Hari and Renvall, 2001) across successive fixations and to disengage in irrelevant noise (e.g., crowding effects; Moores et al., 2011). If the neural processes underlying visual attention are deficient, the development of fluent reading will become difficult. Indeed, visual attention deficits (Hari and Renvall, 2001; Facoetti, 2004, 2012; Valdois et al., 2004; Vidyasagar and Pammer, 2010; Gori and Facoetti, 2014; for reviews) and oculomotor deficits (Pavlidis, 1981; Bucci et al., 2008a,b) have been frequently described in dyslexia. For example, Bosse et al. (2007) reported that the visual span deficit, which was defined as the simultaneous processing of a number of distinct visual elements, was found to account for the reading performance of dyslexics, irrespective of their phonological ability. Bucci et al. (2008a) found that dyslexic readers had an abnormally longer latency for saccades and vergence. Notably, because the visual attention deficit theory has newly been put forward, the neural basis of visual attention deficit for dyslexia has only begun to receive attention in research (e.g., Lobier et al., 2014).

A proposed neurobiological substrate of visual attention deficits in dyslexia could be the dysfunction of the fronto-parietal attentional network (Livingstone et al., 1991; Stein and Walsh, 1997; Gori and Facoetti, 2014). The visual attention deficit theory derives from the magnocellular deficit theory (i.e., developmental dyslexics have a specific deficit in the magnocellular visual system, which spreads from the magno-cells in the retina to the magnocellular layers of the thalamus. Magno-cells are particularly sensitive to low contrasts and moving stimuli with low spatial frequency and play a role in eliminating potential blur due to continuous activation of the sustained parvocellular system during reading. See Stein and Walsh, 1997 for details), but may be more relevant to the extension of the magnocellular system in the dorsal part of the parietal and frontal cortex. fMRI studies using FC analyses have revealed that the regions within the dorsal visual system are strongly and positively correlated (e.g., Fox et al., 2005, 2006). Typically, the intraparietal sulcus (IPS) is known to be a central node for the dorsal visual region in humans (Grefkes and Fink, 2005). The bilateral IPS are consistently activated in eye movement and visual attention tasks (Corbetta et al., 1998; Simon et al., 2002), in line with their function for spatial representation and spatial updating (Merriam et al., 2003; Silver and Kastner, 2009; Pertzov et al., 2011). Such functioning of the IPS could be very important for the visual spatial factors in saccadic fluent reading. Recent studies of task-based fMRI on dyslexia have found the deactivation of their IPS/SPL in visual attention demanding tasks such as multiple element processing (Siok et al., 2009; Peyrin et al., 2011; Lobier et al., 2014). Here, we ask how the IPS interacts with other brain regions to be correlated with fluent reading. The present study focuses on functional disconnections with the central node of the dorsal pathway in dyslexic children.

Of interest, the functions of the dorsal and ventral visual region may not be independent of each other. The right ventral region and the dorsal region have been found to be structurally or functionally connected to each other and to converge in the prefrontal cortex in a study of the neural mechanism for face recognition (e.g., Takahashi et al., 2013). When reading, the left ventral visual region, such as the left occipitotemporal cortex (OTC; McCandliss et al., 2003; Shaywitz and Shaywitz, 2005; Richlan et al., 2009; for reviews), has been consistently found to engage in word processing. In this case, the dorsal visual region may be even more likely to interact with ventral visual region in reading because their corresponding sub-skills for reading (i.e., visual attention and word recognition) are mutually affected (Rayner, 1998; for a review). As increasing emphasis has been placed on integration and interaction of distributed neural systems for complex brain functions, FC analysis has become a useful tool to investigate the inter-regional associations (Friston, 2011). While task-based fMRI studies have provided valuable results for the dyslexics' deficits in FC among reading related regions (e.g., van der Mark et al., 2011; Finn et al., 2014), it is likely influenced by task-induced factors (Friston, 2005; Koyama et al., 2010). Resting state FC, which measures correlations of low-frequency Blood-Oxygenation-Level Dependent (BOLD) signal fluctuations (≈0.01–0.1 Hz) between local areas that are spontaneously activated during rest (Biswal et al., 1995), can explore the brain's intrinsic functional organization and examine if it is altered in neurological or psychiatric diseases (Friston, 2005; Koyama et al., 2010). With this technique, Vogel et al. (2012) found that the visual word form area (VWFA), which is a critical region in the ventral visual region for single word reading (Cohen et al., 2000, 2002; McCandliss et al., 2003), was functionally connected to many dorsal brain regions, including the IPS, the frontal eye field (FEF) and the middle frontal gyrus (MFG). Their participants are adults and typically developing children. Meanwhile, using the left IPS as a seed for resting-state FCs in three groups of dyslexic children (no remediation, partial remediation, and full remediation conditions) and one group of typically developing children, Koyama et al. (2013) found that there was significantly higher FC between the left IPS and the left MFG in the typically developing group relative to the dyslexic groups. Taken together, the relationship between the dorsal and ventral visual regions and their impairments in resting-state fMRI have not been systematically and synchronously explored in dyslexia yet, which is another question addressed in the present study.

In summary, the goals of our investigations are three-fold: (1) to identify the resting-state FC networks in the dorsal and ventral visual regions in children. Based on the findings of previous studies, we chose two central nodes belonging to the dorsal and ventral pathways, the left IPS, and the VWFA, respectively, as seed points for FC analyses; (2) to explore, for the first time, dyslexics' deficits in both the dorsal and ventral visual regions according comparison with the resting-state FC maps for the left IPS and the VWFA between controls and dyslexics; and (3) to reveal the roles of the dorsal and ventral visual regions in fluent reading by correlating the scores of one reading-related task requiring eye movement (i.e., reading fluency) and another reading-related task without overt eye movement (i.e., lexical decision) with the strengths of FCs across groups.

## Material and methods

### Participants

The 47 Chinese children (mean age 12 years ± 1.4 years) who participated in this study were grouped into controls (*n* = 26) and dyslexics (*n* = 21). The children were diagnosed as dyslexic during 4th through 6th grade in primary school if they scored either (1) at least 1.5 standard deviations (*SD*s) below their respective grade mean in the character recognition task or (2) at least 1 *SD* below in the character recognition task and 1.5 *SD*s below in the word list reading task (Zhang et al., 2012; Xue et al., 2013). The diagnosis criteria have been successfully used in studies of dyslexia in China (e.g., Pan et al., 2013). The normal children who participated in our study all scored −0.5 *SD*s above (i.e., 0.5 *SD*s below their respective grade mean at worst; the average score is 0.6 *SD*s above their respective grade mean) in the character recognition task. The dyslexic group also exhibited impaired performance in a battery of reading-related tests described in the study of Xue et al. (2013) (including rapid automatized naming, phoneme deletion, and morphological production), which were completed after MRI data acquisition. Children in each of these two groups were matched by age, sex, handedness, and non-verbal IQ. All of the participants had normal IQ, i.e., above 85 on the Chinese version of the Wechsler Intelligence Scale for Children (C-WISC; Gong and Cai, 1993), or were above the 10th percentile on the Raven's Standard Progressive Matrices (Raven and Court, 1998). The demographic data and scores from the reading-related tests are reported in Table 1.

**Table 1:** **Demographic characteristics (mean ± standard deviation) of the controls and dyslexics and group differences**.

	**Controls**	**Dyslexics**	***t-value***	***p*-value**
*N*	26	21	–	–
Age	12.0 ± 1.2	12.0 ± 1.6	−0.390	n.s.
Sex(male/female)	10/16	12/9	–	n.s.
Handedness(right/left)	26/0	21/0	–	n.s.
Rapid automatized naming(ms)	14 ± 3	18 ± 4	−4.387	*p* < 0.05
Phoneme deletion	22 ± 4	17 ± 5	4.201	*p* < 0.05
Morphological production	25 ± 4	20 ± 4	4.148	*p* < 0.05
Reading fluency(char/min)	408 ± 131	234 ± 121	4.679	*p* < 0.05
Lexical decision(ms)	624 ± 104	687 ± 107	−2.037	*p* < 0.05
Mean frame-wise displacement	0.40 ± 0.02	0.48 ± 0.04	−0.887	n.s

Subjects with a history of neurological diseases or psychiatric disorders were excluded. Except for the 47 valid participants, eight additional children were not included in the following analyses because their head motion exceeded 3 mm. The children and their parents signed informed written consent before the experiment. The study was approved by the Institutional Review Board (IRB) of Beijing Normal University Imaging Center for Brain Research.

### Behavioral tasks of interest

We selected *reading fluency* and *lexical decision* as the behavioral tasks of interest in the present study.

#### Reading fluency

This test was aimed at measuring efficiency in fluent reading. The materials included 100 sentences, gradually increasing in length across the test. Children were given 3 min to silently read as many sentences as possible and to indicate the correctness of the sentence meaning with “
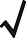
” or “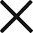.” The score of this task denoted the amount of characters that one can read per minute and was transformed to a z-score (Xue et al., 2013).

#### Lexical decision

This test could measure orthographic awareness. The materials included 200 items (40 for real characters, 40 for non-characters with real radicals in illegal positions, 40 for non-characters with ill-formed components, 40 for scramble strokes filled in one character space, and 40 items as fillers). The children were required to decide whether each stimulus that was presented in the center of the computer screen for 1 s was a real character or not. The reaction time was divided by accuracy to yield a score, and the score was transformed to an inverse number of the *z*-score (Su et al., 2015). One child did not take part in the lexical decision experiment (She set her departure ahead in behavioral test because there is something urgent waiting for her) and thus sustained 46 data points for this task.

Both of these tasks include visual aspects of reading, but they are different in the involvement of visual attention and saccadic eye movements: *reading fluency* requires the subject to read many long sentences with eye movements, whereas *lexical decision* requires the subject to recognize each stimulus that is presented in the center of the computer screen without overt eye movements. Thus, *reading fluency* is more related to visual attention skills compared to *lexical decision* tasks and thus may be more associated with the function of the dorsal visual region. In the following analyses, we concentrated on the relationship between the scores of these two tasks and the strengths of the FCs.

### Imaging acquisitions and data preprocessing

MRI data were obtained on a SIEMENS TRIO 3-Tesla scanner in the Beijing Normal University Imaging Center for Brain Research. We collected resting-state fMRI data using an EPI sequence with the following parameters: 240 EPI functional volumes; 33 axial slices, thickness/gap = 3/0.6 mm, in-plane resolution = 64 × 64, TR = 2000 ms, TE = 30 ms, flip angle = 90°, and FOV = 200 × 200 mm^2^. During the resting-state session, the children were instructed to lie as motionless as possible and not to think systematically.

Image preprocessing was carried out using the Data Processing Assistant for Resting-State fMRI pipeline analysis (DPARSF; Yan and Zang, 2010). For each participant, after converting the DICOM files to NIFTI images, the first 10 time points were discarded to allow for scanner stabilization and the subject's adaptation to the environment. The preprocessing on the remaining time points included: (1) slice timing for interleaved acquisitions, (2) a realigning step to correct for inter-scan head motions, (3) normalization of the functional images into the Montreal Neurological Institute (MNI) space using an echo-planar imaging (EPI) template (Ashburner and Friston, 1999) and resampling to 3 × 3 × 3 mm^3^, (4) spatial smoothing with a 4 mm FWHM Gaussian kernel, (5) removal of the trend of time courses, (6) temporal band-pass filtering (0.01–0.08 Hz), and (7) nuisance correction by regressing out six motion signals as well as individual white matter, cerebrospinal fluid and the global signals. We also explored the possible effects of global signal regression, finding that the results with and without global signal regression were in general similar (see Supplementary Materials for the results without global signal regression). The mean frame-wise displacement was calculated by accounting for head motion at the group-level analysis (Van Dijk et al., 2012), and there was no significant difference in the mean frame-wise displacement between groups [*F*_(1, 46)_ = 0.786, *p* = 0.380].

### FC analyses

The current study focused on the functional networks of the dorsal and ventral visual regions and the relationship between them in the controls and dyslexics. Thus, we defined two representative seed regions for the dorsal and ventral visual pathways to examine their FCs with other areas in the whole brain. The seed for the dorsal pathway centered on the left IPS, which was obtained from a meta-analysis of 18 studies on eye movement (−24, −67, and 40 in MNI coordinates, Brodmann [BA] 7; Jamadar et al., 2013) and the seed for the ventral pathway centered on the VWFA coordinate for Chinese children (−48, −51, and −12 in MNI coordinates, BA 37; Li et al., 2013).

For each subject, the resting-state time course was extracted for 4 mm spheres centered on the VWFA and the left IPS. The regional time course was calculated by averaging the time series of all of the voxels within the seed region. Then, the time course for each of the seed regions was correlated with every other voxel in the brain to generate individual seed maps (Fisher-*r*-to-*z* transformed). Finally, for each seed region, group-level analyses were performed. (1) One-sample *t*-tests for the seed maps of the controls and dyslexics were conducted. Whole-brain correction for multiple comparisons was performed using Gaussian Random Field Theory (Flitney and Jenkinson, 2000; voxel significance: *p* < 0.01, cluster significance: *p* < 0.01). (2) Independent two-sample *t*-tests (voxel significance: *p* < 0.01, cluster significance: *p* < 0.01) for seed maps between the control group and dyslexic children were conducted. The results were visualized using the template surface of smoothed ICBM152 in BrainNet Viewer (Xia et al., 2013).

In addition, we demonstrated that there was an overlapped region in which each voxel was disconnected from both the left IPS and the VWFA. Then, we calculated the correlations (Fisher-*r*-to-*z* transformed) among the time courses in the IPS, the VWFA, and the overlapped MFG region. For illustration, the ROI-wise FCs for each group are shown in bar plots. With respect to the reviewed function of the dorsal and ventral visual regions, we specifically correlated the scores of reading fluency and lexical decision tasks with the strengths of identified FCs across groups. Reading fluency required eye movement, while lexical decision did not, so we expected that the former would be more related to the dorsal region. Sex, age, and the head motion parameter were included as control variables in all group-level analyses.

## Results

### FCs of the left IPS and the VWFA

**Figure 1:**
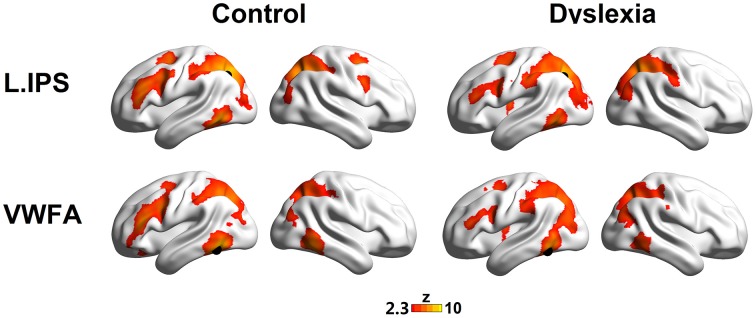
**The left IPS (top panel) and the VWFA (bottom panel) seed maps for the controls (left panel) and dyslexics (right panel)**. The maps display voxels showing significant correlations (voxel *p* < 0.01, cluster *p* < 0.01, corrected) with the time courses of the left IPS and the VWFA. The locations of the seeds are marked with black spheres.

The seed maps presented the areas that had significant FCs with the dorsal visual region (the left IPS) and the ventral visual region (the VWFA) (voxel *p* < 0.01, cluster *p* < 0.01, corrected; see Figure 1 and Table 2).

**Table 2:** **Positively correlated regions defined from the VWFA and the left IPS seed maps**.

	**VWFA seed map**						**Left IPS seed map**					
	**No. of voxels**	**MNI coordinate**			***Z*-score**	**Location**	**No. of voxels**	**MNI coordinate**			***Z*-score**	**Location**
		***X***	***Y***	***Z***				***X***	***Y***	***Z***		
CON	1975	−27	−66	39	6.66	L.IPS	5389	−24	−66	39	11.72	L&R.IPS/L.ITG/L.FG
	1565	−45	30	15	6.58	L.MFG/L.PREC/L.IFG	1209	−39	30	21	6.5	L.MFG/L.PREC
	1297	30	−63	48	5.29	R.IPS	661	27	0	57	5.54	R.MFG/R.PREC
	1194	−48	−51	−12	11.22	L.ITG/L.FG						
	957	51	−51	−12	7.89	R.ITG/R.FG						
DYS	3179	−48	−51	−12	9.55	L.ITG/L.FG/L.IPS	5820	−24	−66	39	10.99	L&R.IPS/L.ITG/L.FG
	2005	30	−60	39	5.86	R.IPS/R.ITG	1184	−27	0	60	5.02	L.MFG/L.PREC
	943	−39	−6	12	4.76	L.MFG/L.PREC/L.IFG						

*CON, controls; DYS, dyslexics; VWFA, visual word fusiform area; IPS, intraparietal sulcus; ITG, inferior temporal gyrus; FG, fusiform gyrus; MFG, middle frontal gyrus; IFG, inferior frontal gyrus; PREC, precentral gyrus*.

The seed maps of the left IPS in both groups revealed a network composed of the bilateral IPS, the left ITG/FG, left MFG, and left FEF. Time courses of the regions in this network were positively correlated with the average time course of the left IPS. In addition, there were also voxels in the right MFG and right FEF for the network in the control group.

The seed maps of the VWFA in both groups were very similar to those of the left IPS, but no voxels survived in the right frontal region. In line with Vogel's et al. (2012) findings, the oscillation of the VWFA in the resting state was positively correlated with dorsal attention areas.

Interestingly, we observed that the left MFG was functionally connected to both the VWFA and the left IPS, suggesting that the left MFG might be a converged region for the dorsal and ventral regions. Numerically, as shown in Figure 1, fewer voxels in the prefrontal regions were observed to correlate with the VWFA and the left IPS in the dyslexic group compared to the control group. We further calculated the superthreshold voxels (i.e., Fisher r-to-z transformed correlation coefficients > 0.2) in the left MFG mask (AAL atlas in Tzourio-Mazoyer et al., 2002) of the individual seed maps, finding that there was marginally significant group effect for the VWFA seed map (*t* = 1.83, *p* = 0.075) and significant group effect for the left IPS seed map (*t* = 2.70, *p* = 0.010).

### Group effects on the FCs of the left IPS and the VWFA

**Figure 2:**
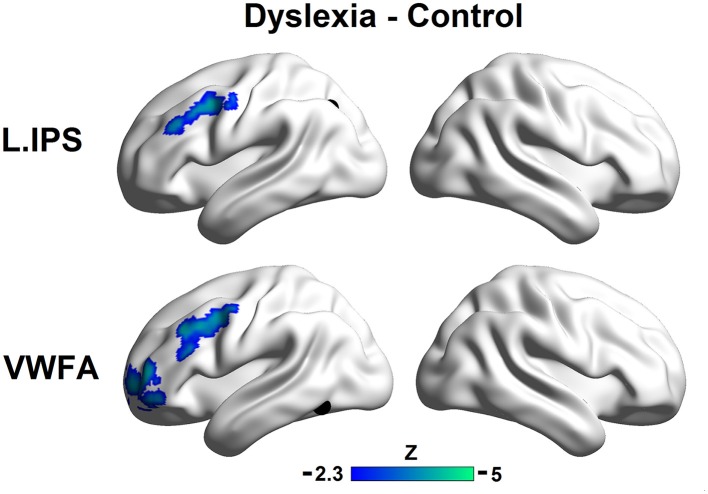
**Group differences in the left IPS (top panel) and the VWFA (bottom panel) seed maps**. The maps display voxels showing significantly reduced FCs with seed regions in the controls relative to the dyslexic subjects (voxel *p* < 0.01, cluster *p* < 0.01, corrected). The locations of the seeds are marked with black spheres.

Direct comparisons of the seed maps between the groups were carried out by independent two-sample *t*-tests (voxel *p* < 0.01, cluster *p* < 0.01, corrected; see Figure 2).

The region showing stronger FC with the left IPS for controls relative to dyslexics included the left MFG (MNI coordinate of peak: −36, 6, 39; *k* = 184; BA 9). Regions showing stronger FCs with the VWFA for controls relative to dyslexics included the anterior part of the left MFG (MNI coordinate of peak: −39, 54, 9; *k* = 258; BA 10) and the left MFG (MNI coordinate of peak: −48, 6, 45; *k* = 240; BA 9). If we conducted the analyses without removing the global signals, the patterns of results in the present study were not affected (see Supplemental Materials for details).

**Figure 3:**
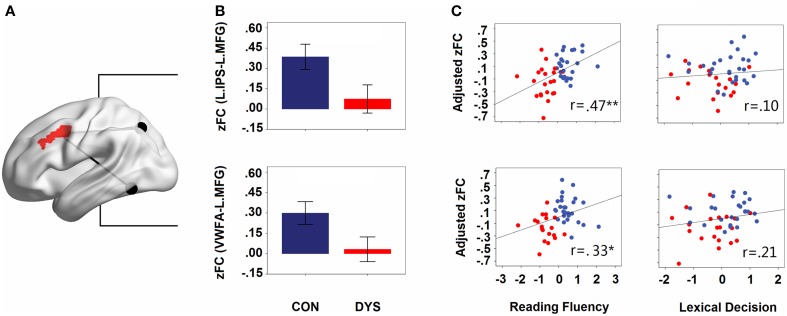
**(A)** The overlapped area (left MFG) of group differences in the left IPS and the VWFA seed maps. The locations of the seeds are marked with black spheres. **(B)** Group differences in the strengths of the FCs (Fisher's *z*-scores of the FCs for IPS- MFG and VWFA-MFG in controls and dyslexics, respectively). Error bars show 95% confidence intervals. **(C)** Partial correlations between the strengths of the FCs (adjusted Fisher's *z*-scores of the FCs for IPS-MFG and VWFA-MFG) and behavioral scores (*z*-scores for lexical decision and reading fluency). CON, controls; DYS, dyslexics; r, coefficient of correlation.

Consistent with the comparative observations, two sample *t*-tests confirmed that dyslexics demonstrated functional alterations not only in the seed map of the VWFA but also in the left IPS, and these two regions were both disconnected to the left MFG. The overlapped area showing group differences between the seed maps of both the left IPS and the VWFA contained 100 voxels in the left MFG (see Figure 3A). In Figure 3B (only for illustration), we presented the results in a ROI-wise manner: while there were very strong FCs of MFG-VWFA and MFG-IPS for controls, these FCs for dyslexics were relatively weak.

### Relationships between FCs and behavioral tasks of interest

When the age, sex, and head motion of subjects were controlled for, the score of reading fluency increased significantly with growing strengths of FCs for the IPS-MFG and the VWFA-MFG couplings (partial *r* = 0.47, *p* = 0.001 and partial *r* = 0.33, *p* = 0.027; see Figure 3C). However, there was no significant correlation between the lexical decision score and the strength of the FC for either the IPS-MFG or the VWFA-MFG (partial *r* = 0.21, *p* = 0.19 and partial *r* = 0.10, *p* = 0.510; see Figure 3C). Additionally, when LD effects were controlled for, the correlation between the reading fluency score and the strength of the FC for the IPS-MFG remained significant (partial *r* = 0.41, *p* = 0.008) and the correlation between the reading fluency score and the strength of the FC for the VWFA-MFG were marginally significant (partial *r* = 0.30, *p* = 0.052).

## Discussion

In the present study, we have shown that the left IPS and the VWFA had a similar FC pattern with regions in the bilateral ITG, IPS, and the left MFG, suggesting that regions for single word reading (i.e., the VWFA) and visual attention (i.e., the IPS) are functionally connected to each other and form a neural circuit together with the left MFG. More importantly, we have identified FC alterations in this neural circuit for dyslexic children: they had weaker strengths of resting-state FCs between the VWFA and the left MFG and between the left IPS and the left MFG relative to the controls. Finally, we observed that the strengths of resting-state FCs between the VWFA and the left MFG and between the left IPS and the left MFG were positively correlated with the reading fluency score, but were not correlated with the lexical decision score, confirming the role of the altered connectivity in fluent reading.

So far, visual attention deficits in dyslexia have been mainly investigated by behavioral studies (Hari and Renvall, 2001; Facoetti, 2004, 2012; Valdois et al., 2004; Vidyasagar and Pammer, 2010; Gori and Facoetti, 2014; for reviews). The traditional neuroimaging studies in the field of visual research tended to examine the neural mechanisms of visual attention with only simple visual stimuli, such as dots or geometric drawings (Corbetta et al., 1998; Simon et al., 2002). However, little is known about the neural mechanisms of visual attention in fluent reading (i.e., saccadic sentence reading) and visual attention deficits in dyslexia, although recent fMRI studies have suggested that the dorsal visual region, the region for visual attention (Corbetta and Shulman, 1998, 2002; Kastner et al., 1999; Simon et al., 2002), may contribute to processing word materials (e.g., Cohen et al., 2008; Lobier et al., 2012). In the present study, with resting state FC analyses, we have further shown the importance of the dorsal visual region in fluent reading and the associated deficits in dyslexia. Compared to recent fMRI studies that reported dyslexics' dysfunction in isolated dorsal regions, such as the IPS/SPL (Siok et al., 2009; Peyrin et al., 2011; Lobier et al., 2014), the V5/MT (compared to age matched controls, Olulade et al., 2013) and the MFG (Siok et al., 2004), our results found altered synchronization among these dorsal areas.

Expressly, we believe that the disconnected dorsal network is specifically related to tasks, such as saccadic reading with overt attention shifting or attention allocation demands. According to neuroimaging studies on visual attention and eye movement using non-alphanumeric materials, the activation of dorsal regions (e.g., the IPS, the FEF, and the MFG; Corbetta and Shulman, 1998, 2002; Kastner et al., 1999; Simon et al., 2002) and cooperation among these dorsal regions (Hwang et al., 2010; Pa et al., 2014) have been consistently observed during classic tasks. To address the function of dorsal visual regions in reading, we correlated the strength of identified FCs between the left IPS and the left MFG with one reading task that requires eye movement and visual attention skills (i.e., fluent reading) and another task that does not require these skills (i.e., lexical decision). The results have shown that the FC between the left IPS and the left MFG was associated with fluent reading even when the lexical decision score was regressed out of the analysis, intensifying the role of the fronto-parietal network in saccadic reading. According to the computation models of eye movement control in reading, attention factors, such as attention shifting (Reichle et al., 2003), attention allocation (Engbert et al., 2005) and parafoveal processing (Rayner, 1975), are critical in both the decisions of eye movement and the processing of words. Further, in behavioral studies, researchers have found visual span deficits (Valdois et al., 2004), attention shift deficits (Facoetti et al., 2000), serial searches and spatial cueing deficits (Franceschini et al., 2012), and eye movement deficits (Bucci et al., 2008a) in dyslexic subjects. It is worth mentioning that our results have revealed the possible neural mechanisms for these behavioral findings.

The most striking findings of the current study are that the dyslexics not only had disconnection within the dorsal visual region (i.e., between the left IPS and the left MFG) but also had disconnection between the ventral and dorsal areas (i.e., between the VWFA and the left MFG), which means that the ventral and dorsal areas of dyslexics are disconnected to the same prefrontal region, known as the left MFG. Recently, Koyama et al. (2013) have found a group difference in the resting state FC between the left MFG and the left IPS, and attributed this result to deficits in the attention network in dyslexics. They also observed differences between occipital areas, and between the right medial prefrontal cortex and fusiform gyrus (FG). While the previous studies have identified part of the altered FCs in the current study, we are the first to use a dual route approach to investigate the dyslexics' deficits in resting state FC, finding that there were dual FCs from the VWFA and the left IPS to the same prefrontal cortex (i.e., the left MFG) in normal children and dual deficits in these two FCs in dyslexic children. These results suggest a systematic deficit in a triangle brain network for the dyslexics. It is worth mention that Vandermosten et al. (2012a,b) have used a dual route approach to investigate the dyslexics' deficits in structural connectivity, finding that fractional anisotropy was different between groups in the left arcuate fasciculus (dorsal phonological route) but not in the inferior frontal-occipital fasciculus (ventral orthographic route). However, whether there is anatomical basis for the visual attention related FC network and its alteration in dyslexics remain to be investigated.

Interestingly, the current identified network is similar to the frontoparietal network that was revealed by ICA or clustering approach in resting state (Cole et al., 2010; Yeo et al., 2011). Relating our current context, the frontoparietal network is a sensory-motor circuit which involves in the saccade task (e.g., Corbetta, 1998). Meanwhile, we have highlighted the cooperation between the VWFA and the frontoparietal network in reading context. While the VWFA is classically viewed as belonging to the ventral visual pathway for computing the visual word representation (McCandliss et al., 2003; Dehaene et al., 2010), recent FC studies (e.g., Vogel et al., 2012; Wang et al., 2015) and our current results have consistently shown that it is strongly functional connected to the dorsal attention regions, suggesting its potential role as an intermediate node for the communication between visual attention processes and word reading processes, such as providing the orthography representation for saccade targeting. However, this proposition needs to be tested explicitly in future studies. As we did not see correlation between lexical decision and the VWFA-MFG connection strength, it is possible that lexical decision can be achieved by the VWFA locally and/or through its connections to other regions whose representations help with lexical decision, but not necessarily with the MFG. This speculation was supported by our additional analysis, where we computed whole brain correlation between the VWFA seed map and the lexical decision score (see details in Supplementary Materials), and found that the isolated lexical decision performances correlated most strongly with FCs around the VWFA and between the VWFA and the angular gyrus.

In fluent reading, the visual attention and word recognition processes influence each other interactively, which implies that language-related ventral areas and attention-related dorsal areas should be studied together. However, while there has been tremendous emphasis on ventral regions in previous fMRI studies of reading and dyslexia, researchers seldom pay attention to how the ventral and dorsal regions cooperate in reading and whether dyslexic individuals have problems with this relationship. According to the results, we speculate that the coordination of the ventral (i.e., the VWFA) and the dorsal (i.e., the left IPS) regions is mediated by the left MFG, which has been reported to be activated mainly in visual attention tasks (McCarthy et al., 1996; Sweeney et al., 1996; Belger et al., 1998; Carlson et al., 1998) but also in some cases of visual word processing (Pugh et al., 2000; Kuo et al., 2004; Tan et al., 2005; Wu et al., 2012). Similarly, Corbetta et al. (2008) also proposed a transmitting function of the MFG: dorsal attention areas, such as the FEF and the IPS, send top-down biases via the MFG to the ventral network, directing ventral activation to behaviorally important stimuli. Heinzle et al. (2010) proposed the switching function of the FEF, which is located slightly superior (but near) to the MFG: there is a global rule that the FEF signals either “reading” or “not reading” and switches the network's behavior from reading to scanning. We have validated these speculations by correlating brain data with behavioral data: the strengths of the two FCs in the left MFG were significantly correlated with the reading fluency score, but were not significantly correlated with the lexical decision score, suggesting that the left MFG may play a role in modulating the task that requires coordination between word recognition and visual attention. It is worth mentioning that these two behavioral tasks had similar variations, so the insignificant correlations between lexical decision scores and brain data were unlikely due to a lack of power. While we have found no effects of lexical decision deficits on the FC between the VWFA and the left MFG, we contend that isolated lexical decisions may be more relevant to the VWFA locally and that the FC between the VWFA and the left MFG probably corresponds to coordination between lexical decisions and visual attention.

Note that the present study has limitations. First, as the amount of semantic processing needed is another difference between reading fluency and lexical decision, it is difficult to distinguish the relatedness to the visual attention or semantic processing for the identified FCs. However, the activation of the IPS was mainly reported in visual attention tasks (Corbetta et al., 1998) in contrast to semantic tasks, suggesting the role of MFG-IPS FC in visual attention. So the current network is at least partly explained by the visual attention aspects of reading. Second, although we have reported behavior-brain relationship in resting state, there was lack of delicate experimental tasks and designs to eliminate other explanations for the function the identified network. Taken together, the results should be interpreted with caution. Future studies can make use of task-based fMRI designs to consolidate our understanding of the relationship between visual attention and fluent reading in dyslexic individuals.

In conclusion, we identified functional disconnections in dyslexic children from the left MFG to both the dorsal visual region (i.e., the left IPS) and the ventral visual region (i.e., the VWFA) in resting state fMRI. These two identified function connections demonstrated positive correlations with reading fluency abilities. The results present the underlying neural mechanism for dyslexics' lack of efficiency in controlling visual attention (e.g., eye movement) while reading scripts.

### Conflict of interest statement

The authors declare that the research was conducted in the absence of any commercial or financial relationships that could be construed as a potential conflict of interest.
