# Supplementary material for: Altered connectivity of the dorsal and ventral visual regions in dyslexic children: a resting-state fMRI study
Source: Front Hum Neurosci. 2015 Sep 10;9:495. doi: 10.3389/fnhum.2015.00495 (PMC4564758; doi:10.3389/fnhum.2015.00495)
Supplement: Supplementary file 1 [file Presentation1.PDF]

Supplementary materials

Figure 1s

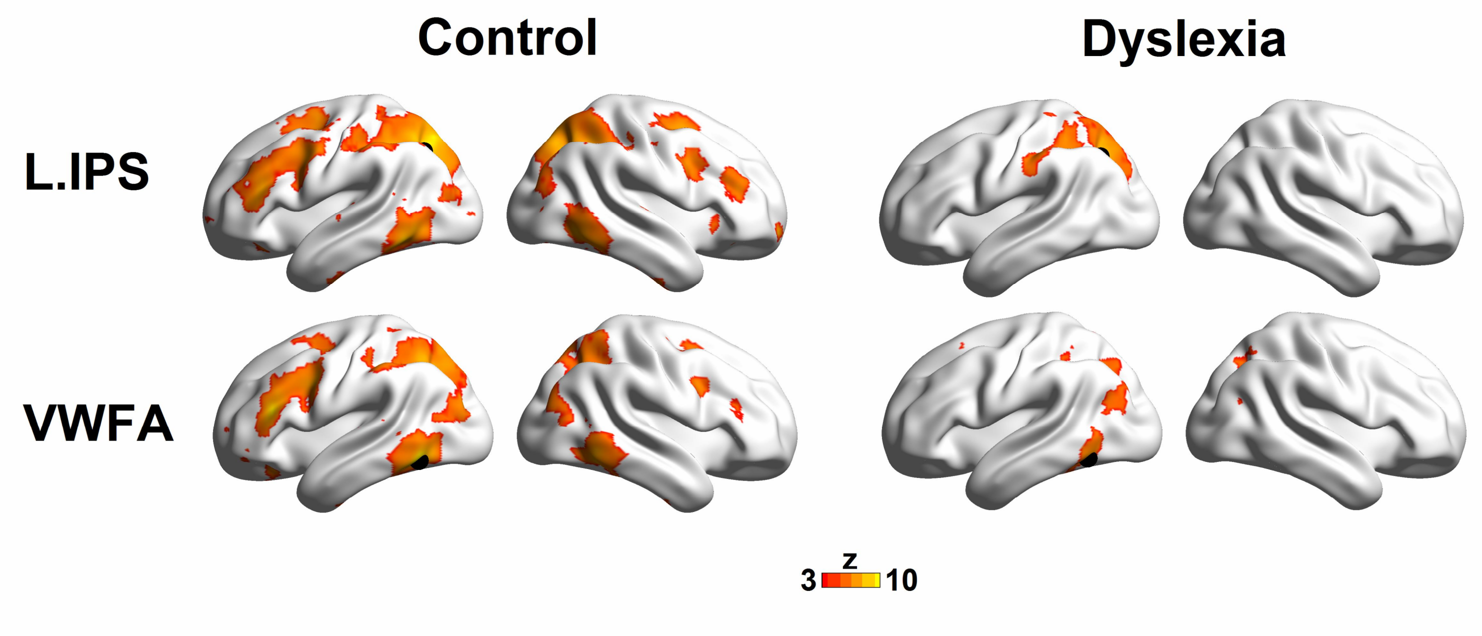

Figure 1s. The left IPS (top panel) and the VWFA (bottom panel) seed maps for controls (left panel) and dyslexics (right panel) ***without removing global signal***. Maps display voxels showing significant correlations (voxel $p < .10^{-8}$, cluster $p < .01$, corrected) with the time courses of the left IPS and VWFA. The locations of the seeds are marked with black spheres.

Figure 2s

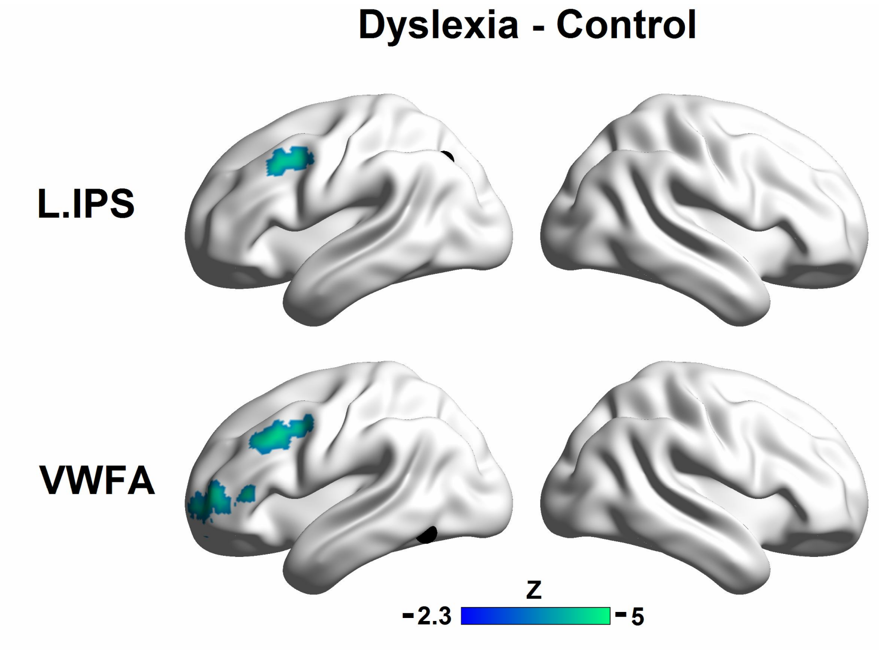

Figure 2s. Group differences in the left IPS (top panel) and the VWFA (bottom panel) seed maps ***without removing global signal***. Maps displays voxels showing significant reduced FCs with seed regions for controls than dyslexia (voxel $p < .01$, cluster $p < .05$, corrected). The locations of the seeds are marked with black spheres.

Figure 3s

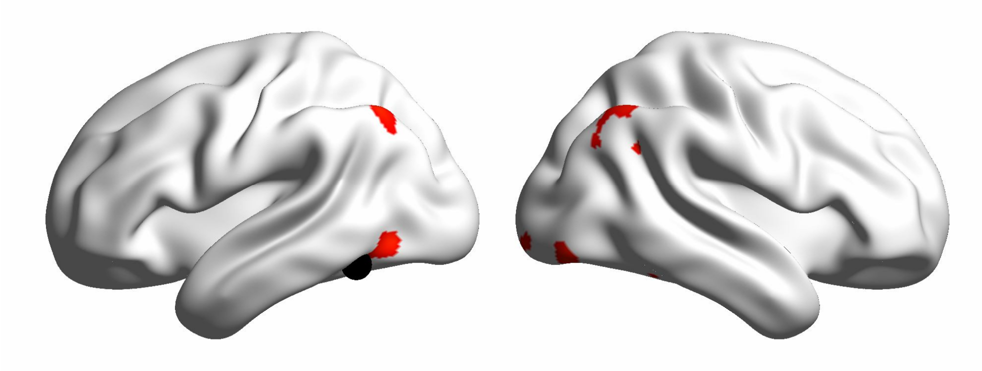

Figure 3s. The result for whole brain correlation between the VWFA seed map and the lexical decision score (voxel level $p < .05$, cluster level $p < .05$) . The locations of the seeds are marked with black spheres.
